# Supplementary material for: Synchronous multi-color laser network with daily sub-femtosecond timing drift
Source: Sci Rep. 2018 Aug 9;8:11948. doi: 10.1038/s41598-018-30348-2 (PMC6085312; doi:10.1038/s41598-018-30348-2)
Supplement: Supplementary file 1 — Supplementary Information [file 41598_2018_30348_MOESM1_ESM.docx]

**Supplementary Information –**

Synchronous multi-color laser network with daily sub-femtosecond timing drift

Kemal Şafak,^1,2^ Ming Xin,^1,3,*^ Michael Y. Peng,^3^ and Franz X. Kärtner,^1,2,3,4^

*^1^Center for Free-Electron Laser Science, Deutsches Elektronen-Synchrotron, Notkestrasse 85, Hamburg 22607, Germany*

*^2^Physics Department, University of Hamburg, Luruper Chaussee 149, 22761 Hamburg, Germany*

*^3^Research Laboratory of Electronics, Massachusetts Institute of Technology, Cambridge, Massachusetts 02139, USA*

*^4^The Hamburg Center for Ultrafast Imaging, Luruper Chaussee 149, 22761 Hamburg, Germany*

**Corresponding author:* [*xinm@mit.edu*](mailto:xinm@mit.edu)

This document provides supplementary information to “Synchronous multi-color laser network with daily sub-femtosecond timing drift”. First, we derive the timing error in a stabilized fiber link network observed due to uncompensated beam paths and frequency noise of the master laser. Then we detail the timing jitter analysis of the laser network described in the main article.

Timing error in a stabilized fiber link network

**Figure S1.** Schematic of a timing-stabilized fiber link network. Abbreviations: M: mirror; PBS: polarization beam splitter; FC: fiber coupler; TD: timing detector; PRM: partially reflecting mirror. TDs are symbolized with “lock symbols”. Sealed lock refers to an in-loop detector; whereas open lock corresponds to an out-of-loop detector.

Here, we derive the timing error in a fiber link network due to uncompensated beam paths and deviation of the master laser’s repetition rate. As a case study, we examine the setup shown in Fig. S1 where the output of a master laser is split to stabilize two fiber links. There is a partially reflecting mirror at the end of each link providing signal for timing measurements both at the master laser location and the link end station. Transmission delay of each link is stabilized with a timing detector (TD), which measures the timing fluctuations of the round-trip traveled pulses with respect to the new laser pulses and applies a feedback control on the variable delay in the link. Once both of the links are stabilized, an out-of-loop TD detects the relative timing error between the two output pulse trains. Suppose that every *m^th^* pulse from the master laser and every (*m*−*m_1_)^th^* link-traveled pulse meet with each other at TD1 (*m* and *m_1_* are integers). Then the measured timing error by TD1 is:

|  | $\Delta t_{TD1}=\frac{m_{1}}{f_{rep}}-\frac{2}{c}(d_{11}-L_{1})$ | (S1) |
| --- | --- | --- |

where *f_rep_* is the repetition rate of the master laser, *c* is the speed of light in vacuum, *d_11_* is the uncompensated reference arm of fiber link 1 (i.e., the distance from PBS1 to M1: *d_11_* = |PBS1 M1|). *L_1_* is the optical path from PBS1 to PRM1 (i.e., *L_1_ =* |PBS1 FC1| + *n*|FC1 PRM1| where *n* is the refractive index of the fiber link). Similarly, every *m^th^* pulse coincides with every *(m*−*m_2_)^th^* pulse (*m_2_* is an integer) at TD2:

|  | $\Delta t_{TD2}=\frac{m_{2}}{f_{rep}}-\frac{2}{c}(d_{21}-L_{2})$ | (S2) |
| --- | --- | --- |

where *d_21_* is the uncompensated reference arm of the fiber link 2 (i.e., *d_21_* = |PBS2 M2|), and *L_2_* is the optical path from PBS2 to PRM2 (i.e., *L_2_ =* |PBS2 FC2| + *n*|FC2 PRM2|). Once the links are stabilized, the feedback loops keep the timing error of each link at zero, i.e. *∆t_TD1_*=*∆t_TD2_*=*0:*

|  | $\frac{m_{1}}{f_{rep}}=\frac{2}{c}(d_{11}-L_{1})$ | (S3) |
| --- | --- | --- |
|  | $\frac{m_{2}}{f_{rep}}=\frac{2}{c}(d_{21}-L_{2})$ | (S4) |

Then, we can observe the out-of-loop timing error between the *(m*−*m_3_)^th^* pulse from fiber link 1 and the *(m*−*m_4_)^th^* pulse from fiber link 2 using TD3 (*m_3_*, *m_4_* are integers). Suppose that at the beginning of the measurement, the out-of-loop TD3 is aligned so that its output shows zero timing error, i.e., *∆t*=*0*:

|  | $\Delta t=\frac{\left( m_{3}-m_{4} \right)}{f_{rep}}-\frac{1}{c}\left[ L_{2}+d_{22}-L_{1}-d_{12} \right]=0$ | (S5) |
| --- | --- | --- |

*d_12_* and *d_22_* are the uncompensated optical paths at the end of fiber link 1 and fiber link 2 respectively, i.e., *d_12_*= |PRM1 M3|+|M3 PBS3| and *d_22_*= |PRM2 PBS3|. Then, if the repetition rate of the master laser changes by some amount of *∆f_rep_*, the new period of the pulse train will be *1/(f_rep_*+*∆f_rep_).* To keep the links stabilized, the in-loop detectors TD1 and TD2 will adjust the variable delays by *∆L_1_* and *∆L_2_* so that *∆T_TD1_*=*∆T_TD2_*=*0.* Then, Eqs. (S3) and (S4) yield:

|  | $\frac{m_{1}}{f_{rep}+\Delta f_{rep}}=\frac{2}{c}(d_{11}-L_{1}-\Delta L_{1})$ | (S6) |
| --- | --- | --- |
|  | $\frac{m_{2}}{f_{rep}+\Delta f_{rep}}=\frac{2}{c}(d_{21}-L_{2}-\Delta L_{2})$ | (S7) |

However, the out-of-loop detector will detect a timing error in this case:

|  | $\Delta t=\frac{\left( m_{3}-m_{4} \right)}{f_{rep}+\Delta f_{rep}}-\frac{1}{c}[L_{2}+\Delta L_{2}+d_{22}-L_{1}-\Delta L_{1}-d_{12}]$ | (S8) |
| --- | --- | --- |

Then by solving Eq. (S8) together with Eqs. (S3) – (S7), we obtain:

|  | $\Delta t=\frac{\Delta L_{2}}{{(L}_{2}-d_{21})c}(d_{21}+d_{22}-d_{11}-d_{12})$ | (S9) |
| --- | --- | --- |

In the experiment, total link length is in the km-scale whereas the reference paths are in the cm-scale, i.e., *L_2_*>>*d_21_*. Furthermore, the ratio of *∆L_2_/L_2_* must be equal to *∆f_rep_/f_rep_* to keep the fiber link stabilized. Hence, the timing error between the two stabilized fiber links will be:

|  | $\Delta t=\frac{\Delta f_{rep}}{f_{rep}}\frac{\Delta d}{c}$ | (S10) |
| --- | --- | --- |

where *∆d=d_21_+d_22_*−*d_11_*−*d_12_* is the length difference of the uncompensated beam paths. Equation (S10) suggests that even if we can eliminate all noise sources in the fiber links and lock the remote slave lasers perfectly to the link outputs, we will still observe a timing error at the output of the synchronous mode-locked laser network due to the frequency noise of the master laser and uncompensated beam paths. Free-running timing jitter of the low-noise mode-locked lasers can add up to picoseconds when integrated down to 1 Hz offset frequency^S1^. For a master laser operating at 200-MHz repetition rate, a pulse period drift (i.e., $\Delta T_{rep}$) of 1 ps will result in a repetition rate shift of 40 kHz (i.e., $\Delta f_{rep}\approx f_{rep}^{2}\Delta T_{rep}$). Even if one can align and stabilize the length of uncompensated beam paths with 1-cm precision (i.e., *∆d* =1 cm), there will be still a timing error of ~7 fs through Eq. (S10). Therefore, the repetition rate of the master laser must be locked to a stable reference and the length of uncompensated beam paths must be minimized in order to achieve sub-femtosecond timing distribution.

Detailed timing jitter analysis

**Figure S2.** Feedback flow diagrams of (a) timing link stabilization, (b) remote slave laser synchronization, and (c) out-of-loop jitter characterization between two remote slave lasers. Subscripts *Li* and *Si* after each abbreviation refer to timing link 1 and slave laser 1 for *i=1* and to timing link 2 and slave laser 2 for *i=2*. Abbreviations: *J_M_* free-running jitter of the master, *J_Si_*: free-running jitter of the slave lasers 1 & 2; *H_BOC_*, *H_BPD_*, *H_PI_* and *H_PZT_*: transfer functions of the BOC, BPD, PI controller and PZT, respectively; *E_BOC_* and *E_PI_*: electronic noise introduced by the BOC and the PI controller; *J_E_*: total environmental jitter imposed on the link for one-way travel; *J_I,_*: detected timing jitter by the in-loop BOC; *J_F_*: equivalent jitter generated by the feedback loop to maintain the lock; *J_out_*: out-of-loop jitter between the two slave lasers detected by SF-BOC; *τ*: one-way fiber link travel time, *s=jω*: complex frequency.

In this section, we develop a comprehensive feedback model to derive the noise sources present in the laser network and their complex jitter transfer functions presented in the main text. The model is adapted from our previous analysis published in ^S2^. The network consists of a master laser and two timing-stabilized fiber links which remotely synchronize two slave lasers to the master laser. Stabilization of the links and synchronization of the remote lasers are performed by locking schemes based on BOCs whose feedback flow diagrams are shown in Fig. S2(a) and S2(b), respectively. Each BOC lock has two electronic noise sources *E_BOC_* and *E_PI_* (in units of V/√Hz) and four transfer functions *H_BOC_*, *H_BPD_*, *H_PI_* and *H_PZT_*. *H_BOC_* is simply the timing sensitivity of the BOC converting the timing error to a voltage signal. The BOC voltage is amplified by the transimpedance amplifier of the balanced photodetector (BPD) with a transfer function *H_BPD_* and processed by the PI controller with a transfer function *H_PI_*. Then, the output of the PI controller is applied to the PZT actuator with a resonance frequency at *f_res_* and a transfer function *H_PZT_*, which converts the voltage signal into a corrective time delay and closes the feedback loop. Using the feedback flow diagram in Fig. S2(a), we obtain the in-loop jitter of the timing links:

|  | $J_{I,Li}=J_{M}\left[ -1+exp \left( -2s\tau_{Li} \right) \right]+J_{E,Li}\left[ 1+\exp\left( -s\tau_{Li} \right) \right]+J_{F,Li}\left[ 1+\exp\left( -2s\tau_{Li} \right) \right]$ | (S11) |
| --- | --- | --- |

together with a term arising from the feedback loop of the link-stabilization:

|  | $J_{F,Li}=\left[ -\left( J_{I,Li}H_{BOC,Li}+E_{BOC,Li} \right)H_{BPD,Li}H_{PI,Li}+E_{PI,Li} \right]H_{PZT,Li}$ | (S12) |
| --- | --- | --- |

where the subscripts *Li* refer to timing link 1 for *i=1* and to timing link 2 for *i=2*. From Fig. S2(b), we determine the in-loop jitter between the slave lasers and the timing links:

|  | $J_{I, Si}=J_{M}\exp\left( -s\tau_{Li} \right)-J_{Si}+J_{E,Li}+J_{F,Li}\exp\left( -s\tau_{Li} \right)+J_{F,Si}$ | (S13) |
| --- | --- | --- |

together with a term arising from the feedback loop of the laser synchronization:

|  | $J_{F,Si}=\left[ -\left( J_{I,Si}H_{BOC,Si}+E_{BOC,Si} \right)H_{BPD,Si}H_{PI,Si}+E_{PI,Si} \right]H_{PZT,Si}$ | (S14) |
| --- | --- | --- |

Subscripts *Si* refer to slave laser 1 for *i=1* whose *f_rep_* locked to timing link 1*;* whereas for *i=2, Si* refer to slave laser 2 whose *f_rep_* locked to timing link 2. For the abbreviations of each specific term, please refer to the caption of Fig. S2. From the diagram in Fig. S2(c), the timing jitter measured by the out-of-loop detector *J_out_* is simply:

|  | $J_{out}=J_{I,S2}-J_{I,S1}$ | (S15) |
| --- | --- | --- |

To simplify the mathematical derivation, we define “electronic noise” terms *J_N,j_* and “total transfer functions” *H_j_* for each BOC setup as:

|  | $J_{N,j}=-\frac{E_{BOC,j}}{H_{BOC, j}}+\frac{E_{PI,j}}{H_{BOC,j}H_{BPD,j}H_{PI,j}}$ | (S16) |
| --- | --- | --- |
|  | $H_{j}=H_{BOC,j}H_{BPD,j}H_{PI,j}H_{PZT,j}$ | (S17) |

Subscripts *j=L1, L2, S1* and *S2* refer to the specific BOC-locks stabilizing timing link 1 and 2, and synchronizing slave laser 1 and 2, respectively. By solving the Eqs. (S11) – (S15) together and using the definitions in Eqs. (S16) and (S17), the out-of-loop timing jitter will be:

|  | $J_{out}=C_{M}J_{M}+C_{S1}J_{S1}+C_{S2}J_{S2}+C_{E,L1}J_{E,L1}+C_{E,L2}J_{E,L2}+C_{N,S1}J_{N,S1}+C_{N,S2}J_{N,S2}+C_{N,L1}J_{N,L1}+C_{N,L2}J_{N,L2}$ | (S18) |
| --- | --- | --- |

There are nine uncorrelated noise sources at the out-of-loop timing detector: inherent timing jitter of the master laser (*J_M_*) and the slave lasers (*J_S1_* and *J_S2_*), the environmental noise imposed on the timing links (*J_E,L1_* and *J_E,L2_*), and the electronic noise of each BOC-lock (*J_N,S1_*, *J_N,S2_*, *J_N,L1_* and *J_N,L2_*). The terms in front of each jitter source are frequency-dependent complex jitter transfer functions which determine the contribution of each noise sources to the out-of-loop timing jitter of the laser network. They are given as:

|  | $C_{M}=\frac{\exp\left( -s\tau_{L2} \right)}{1+H_{S2}}\frac{\left( 1+2H_{L2} \right)}{1+H_{L2}\left( 1+\exp\left( -2s\tau_{L2} \right) \right)}-\frac{\exp\left( -s\tau_{L1} \right)}{1+H_{S1}}\frac{\left( 1+2H_{L1} \right)}{1+H_{L1}\left( 1+\exp\left( -2s\tau_{L1} \right) \right)}$ | | (S19) | |
| --- | --- | --- | --- | --- |
|  | $C_{S1}=\frac{1}{1+H_{S1}}$ | | (S20) | |
|  | $C_{S2}=-\frac{1}{1+H_{S2}}$ | | (S21) | |
|  | | $C_{E,L1}=-\frac{1}{1+H_{S1}}\frac{1+H_{L1}\left[ 1-\exp\left( -s\tau_{L1} \right) \right]}{1+H_{L1}\left[ 1+\exp\left( -2s\tau_{L1} \right) \right]}$ | | (S22) |
|  | | $C_{E,L2}=\frac{1}{1+H_{S2}}\frac{1+H_{L2}\left[ 1-\exp\left( -s\tau_{L2} \right) \right]}{1+H_{L2}\left[ 1+\exp\left( -2s\tau_{L2} \right) \right]}$ | | (S23) |
|  | | $C_{N,S1}=-\frac{H_{S1}}{1+H_{S1}}$ | | (S24) |
|  | | $C_{N,S2}=\frac{H_{S2}}{1+H_{S2}}$ | | (S25) |
|  | | $C_{N,L1}=-\frac{\exp\left( -s\tau_{L1} \right)}{1+H_{S1}}\frac{H_{L1}}{1+H_{L1}\left[ 1+\exp\left( -2s\tau_{L1} \right) \right]}$ | | (S26) |
|  | | $C_{N,L2}=\frac{\exp\left( -s\tau_{L2} \right)}{1+H_{S2}}\frac{H_{L2}}{1+H_{L2}\left[ 1+\exp\left( -2s\tau_{L2} \right) \right]}$ | | (S27) |

All jitter sources in Eq. (S18) have the units of s/√Hz. Since these noise sources are uncorrelated with each other, the average values of their cross products will be zero. Hence, the average out-of-loop jitter spectral density (in units of s^2^/Hz) is:

|  | $\overline{J_{out}^{2}}=\left\vert C_{M} \right\vert^{2}\overline{J_{M}^{2}}+\left\vert C_{S1} \right\vert^{2}\overline{J_{S1}^{2}}+\left\vert C_{S2} \right\vert^{2}\overline{J_{S2}^{2}}+\left\vert C_{E,L1} \right\vert^{2}\overline{J_{E,L1}^{2}}+\left\vert C_{E,L2} \right\vert^{2}\overline{J_{E,L2}^{2}}+\left\vert C_{N,S1} \right\vert^{2}\overline{J_{N,S1}^{2}}+\left\vert C_{N,S2} \right\vert^{2}\overline{J_{N,S2}^{2}}+\left\vert C_{N,L1} \right\vert^{2}\overline{J_{N,L1}^{2}}+\left\vert C_{N,L2} \right\vert^{2}\overline{J_{N,L2}^{2}}$ | (S28) |
| --- | --- | --- |

All terms in Eq. (S28) can be either measured or calculated using the experimental parameters. Since the master laser and the slave laser 1 are identical, we use the experimental data set from our former publication^S3^ for both *J_M_* and *J_S1_* (i.e., *J_M_*=*J_S1_*). We obtain the environmental noise imposed on the links (*J_E,L1_* and *J_E,L2_*) experimentally by measuring the free-running outputs of SH-BOC1 and SH-BOC2 with a baseband analyzer when their feedback loops are disengaged.

The electronic noise of a BOC (*E_BOC_*) is measured (in units of V/√Hz) when there is no input light on the device, and then converted to timing jitter (s/√Hz) by multiplying with the timing sensitivity. The electronic noise of a PI controller (*E_PI_*) is determined from its input voltage noise (*V_n_*) using a servo-control model based on an integrating-operational-amplifier as in ^S2^. We use the following transfer functions for BOC, BPD, PI controller and PZT:

|  | $H_{BOC}=k_{BOC}$ | (S29) |
| --- | --- | --- |
|  | $H_{PI}=k_{PI}\left( \frac{s+2\pi f_{PI}}{s} \right)$ | (S30) |
|  | $H_{BPD}=\left( 1+\frac{s}{2\pi f_{BW}} \right)^{-1}$ | (S31) |
|  | $H_{PZT}=\frac{k_{PZT}}{sf_{rep}}\left( \frac{\left( 2\pi f_{res} \right)^{2}}{s^{2}+\zeta s+\left( 2\pi f_{res} \right)^{2}} \right)$ | (S32) |

where *s=jω* is the complex frequency, *k_BOC_* is the timing sensitivity of the BOC, *k_PI_* and *f_PI_* are the gain and corner frequency of the PI controller. Furthermore, *f_BW_* is the 3-dB bandwidth of the BPD, *k_PZT_* is the slave laser’s PZT sensitivity, *f_rep_* is the repetition rate of the slave laser, *f_res_* is the slave laser’s PZT resonance and *ζ* is the PZT response parameter. All these parameters are noted during the experiments and given in the Table S1. Then, the electronic noise terms (*J_N,S1_*, *J_N,S2_*, *J_N,L1_*, *J_N,L2_*) and the jitter transfer functions (*C_M_*, *C_S1_*, *C_S2_*, *C_E,L1_*, *C_E,L2_*, *C_N,S1_*, *C_N,S2_*, *C_N,L1_*, *C_N,L2_*) are calculated as derived in Eqs. (S16), (S17), (S19) – (S27).

Finally, we estimate the free-running timing jitter of slave laser 2 using the measurement results of the local synchronization given in Fig. 3(b) of the main text (see the red curve). The feedback flow diagram of the local synchronization is very similar the one in Fig. S2(b) except that there is no timing link transmission and stabilization (i.e., *J_E,L2_=J_F,L2_=τ_L2_=0*). By adapting Eq. (S13), we obtain the in-loop jitter of the local synchronization *J_I,S1-S2_*:

|  | $J_{I,S1-S2}=J_{S1}-J_{S2}+J_{F,S1-S2}$ | (S33) |
| --- | --- | --- |

where the term *J_F,S1-S2_* arises from the feedback loop of the local synchronization:

|  | $J_{F,S1-S2}=\left[ -\left( J_{I,S1-S2}H_{BOC,S1-S2}+E_{BOC,S1-S2} \right)H_{BPD,S1-S2}H_{PI,S1-S2}+E_{PI,S1-S2} \right]H_{PZT,S1-S2}$ | (S34) |
| --- | --- | --- |

By defining the electronic noise term *J_N,S1-S2_* and the total transfer function *H_S1-S2_* according Eqs. (S16) and (S17), we can get the local jitter between the two lasers as:

|  | $J_{I,S1-S2}=\left( \frac{1}{1+H_{S1-S2}} \right)\left( J_{S1}-J_{S2} \right)+\left( \frac{H_{S1-S2}}{1+H_{S1-S2}} \right)J_{N,S1-S2}$ | (S35) |
| --- | --- | --- |

Since *J_S1_, J_S2_* and *J_N,S1-S2_* are uncorrelated noise sources with respect to each other, the mean values of their cross products will be zero. Then, we find the average jitter spectral density between the two lasers measured by SF-BOC2 as:

|  | $\overline{J_{I,S1-S2}^{2}}=\left\vert\frac{1}{1+H_{S1-S2}} \right\vert^{2}\left( \overline{J_{S1}^{2}}+\overline{J_{S2}^{2}} \right)+\left\vert\frac{H_{S1-S2}}{1+H_{S1-S2}} \right\vert^{2}\overline{J_{N,S1-S2}^{2}}$ | (S36) |
| --- | --- | --- |

From this equation, we can derive the free running jitter of slave laser 2 as:

|  | $\overline{J_{S2}^{2}}=\left\vert1+H_{S1-S2} \right\vert^{2}\overline{J_{I,S1-S2}^{2}}-\overline{J_{S1}^{2}}-\left\vert H_{S1-S2} \right\vert^{2}\overline{J_{N,S1-S2}^{2}}$ | (S37) |
| --- | --- | --- |

The free-running jitter of slave laser 1 is identical to the master laser (i.e., *J_S1_=J_M_*) and has been already determined. The electronic noise *J_N,S1-S2_* has also been measured and shown with the gray curve in Fig. 3(b) of the main text. The total transfer function *H_S1-S2_* is estimated using the experimental parameters shown in Table S2 and the transfer functions of the experimental equipment given in Eq. (S29) – (S32). Since, all terms on the right-hand side of Eq. (S37) are known; we can determine the free running jitter of slave laser 2.

Fig. S3 shows the timing jitter spectral densities of the noise sources given in Eq. (S28). Slave laser 2 has the highest noise with a free-running timing jitter of 330 fs RMS when the Fig. S3(c) integrated from 100 Hz up to 1 MHz; whereas the electronic noise sources are the lowest proving the high SNR provided by the BOCs used in the systems.


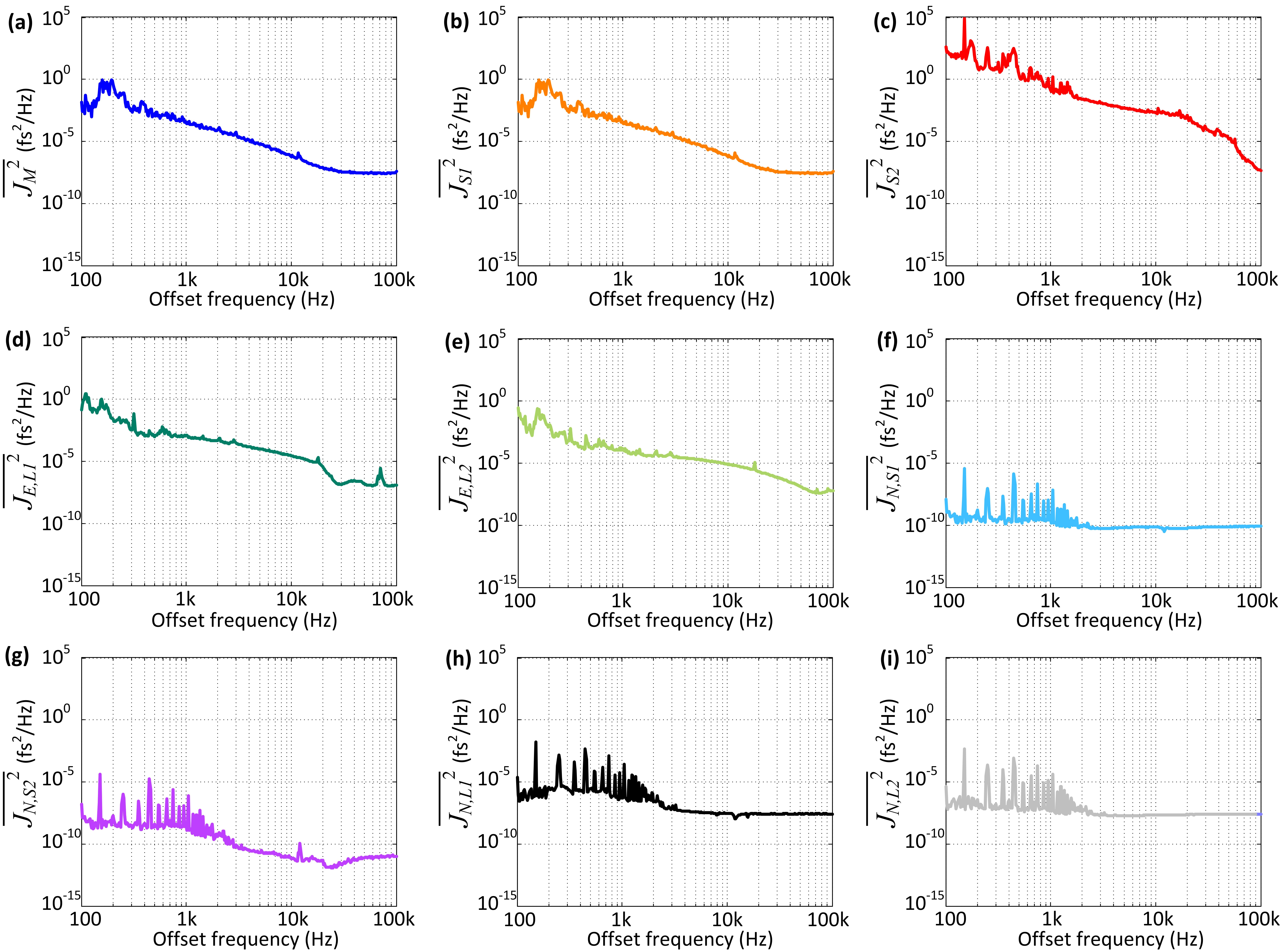


**Figure S3.** Noise sources present in the laser network. Inherent timing jitter of (a) the master laser; (b) slave laser 1 and (c) slave laser 2; environmental noise of (d) timing link 1 and (e) timing link 2; electronic noise arising from the feedback loops of (f) slave laser 1 and (g) slave laser 2, and from the stabilization of (h) timing link 1 and (i) timing link 2. All graphs have the same scale for better comparison.


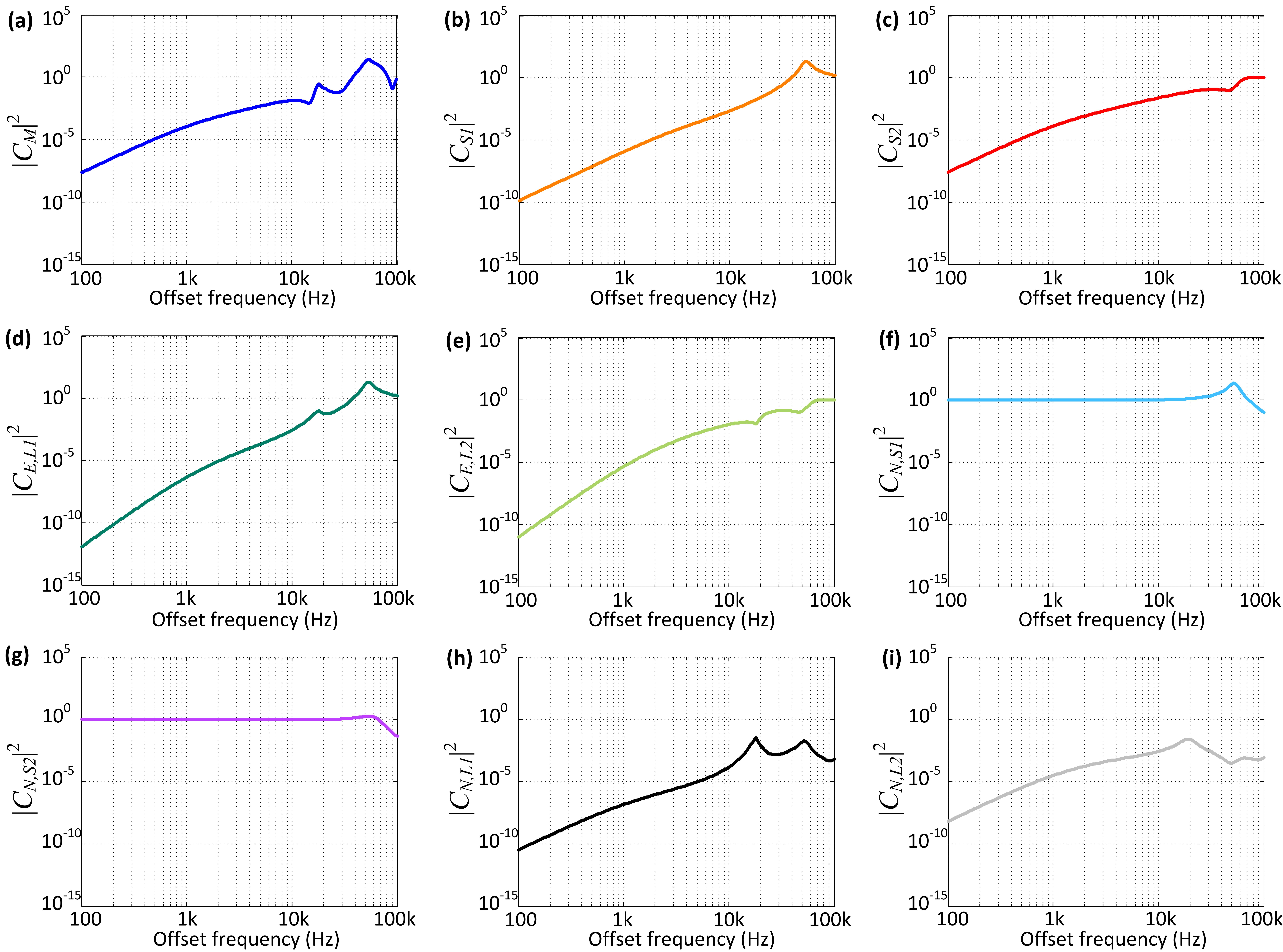


**Figure S4.** Jitter transfer functions of the noise sources shown in Fig. S3. All graphs have the same scale for better comparison.

Finally, Fig. S4 shows the jitter transfer functions of all noise sources assigning their contribution to the output of the laser network. Only a small fraction of the master laser’s noise transferred to the network output due to the symmetry in the synchronization (see Fig. S4(a)). This can be also inferred from the two cancelling terms of Eq. (S19) suggesting that *C_M_* would yield zero if both links, the slave lasers and their feedback loops were identical. Similarly, all other noise sources in the laser network are highly suppressed for offset frequencies less than 20 kHz, except the electronic noise of the two remote laser synchronizations (i.e., Figs. S4(f) and (g)) which are carried straight to the out-of-loop jitter by their feedback loops.

Table S1 and S2 show the experimental parameters used in the calculations for the feedback models presented in the paper.

Table S1: Experimental parameters of the synchronous laser network used for the calculations in Figs. 4, S3 and S4.

| Parameter: | Value: | Unit: |
| --- | --- | --- |
| *k_SH-BOC1_* | 0.93 | mV/fs |
| *k_SH-BOC2_* | 1.41 | mV/fs |
| *k_SH-BOC3_* | 5.00 | mV/fs |
| *k_SF-BOC1_* | 10.00 | mV/fs |
| *k_SF-BOC2_* | 14.95 | mV/fs |
| *k_PI,SH-BOC1_* | 0 | dB |
| *k_PI,SH-BOC2_* | 0 | dB |
| *k_PI,SH-BOC3_* | 15 | dB |
| *k_PI,SF-BOC1_* | 22 | dB |
| *f_PI, SH-BOC1_* | 3 | kHz |
| *f_PI, SH-BOC2_* | 10 | kHz |
| *f_PI, SH-BOC3_* | 3 | kHz |
| *f_PI, SF-BOC1_* | 1 | kHz |
| *f_BW, SH-BOC1_* | 100 | MHz |
| *f_BW, SH-BOC2_* | 100 | MHz |
| *f_BW, SH-BOC3_* | 4 | MHz |
| *f_BW, SF-BOC1_* | 4 | MHz |
| *τ_L1_* | 17.2 | μs |
| *τ_L2_* | 5.8 | μs |
| *k_PZT,S1_* | 14.24 | Hz/V |
| *k_PZT,S2_* | 1.2 | Hz/V |
| *f_Res,S1_* | 40 | kHz |
| *f_Res,S2_* | 50 | kHz |
| *ζ_S1_* | 10^6^ | - |
| *ζ_S2_* | 10^5^ | - |
| *f_rep,S1_* | 216.67 | MHz |
| *f_rep,S1_* | 54.167 | MHz |
| *V_n_* | 10^-9^ | V/√Hz |

Table S2: Experimental parameters of the local synchronization between the slave lasers used for the calculation in Fig. S3(c).

| Parameter: | Value: | Unit: |
| --- | --- | --- |
| *k_SF-BOC2_* | 14.95 | mV/fs |
| *k_PI,S1-S2_* | 2 | dB |
| *f_PI, S1-S1_* | 3 | kHz |
| *f_BW, SF-BOC2_* | 4 | MHz |
| *k_PZT,S2_* | 1.2 | Hz/V |
| *f_res,S2_* | 50 | kHz |
| *ζ_S2_* | 10^5^ | - |
| *f_rep,S2_* | 54.167 | MHz |
| *V_n_* | 10^-9^ | V/√Hz |

References

S1. Jung, K. & Kim, K. Characterization of timing jitter spectra in free-running mode-locked lasers with 340 dB dynamic range over 10 decades of Fourier frequency. *Opt. Lett.* **40**, 316-319 (2015).

S2. Şafak, K. et al. Jitter analysis of timing-distribution and remote-laser synchronization systems. *Opt. Express* **24**, 21752-21766 (2016).

S3. Şafak, K., Xin, M., Callahan, P. T., Peng, M. Y. & Kärtner, F. X. All fiber-coupled, long-term stable timing distribution for free-electron lasers with few-femtosecond jitter. *Struct. Dyn.* **2**, 041715 (2015).
